# Critical thinking: concept analysis from the perspective of Rodger's evolutionary method of concept analysis

**DOI:** 10.1590/1518-8345.1191.2785

**Published:** 2016-09-01

**Authors:** Fábio da Costa Carbogim, Larissa Bertacchini de Oliveira, Vilanice Alves de Araújo Püschel

**Affiliations:** 1 Doctoral Student , Escola de Enfermagem, Universidade de São Paulo, São Paulo, SP, Brazil. Assistant Professor, Departamento de Enfermagem Aplicada, Universidade Federal de Juíz de Fora, Juiz de Fora, MG, Brazil.; 2 Doctoral Student, Escola de Enfermagem, Universidade de São Paulo, São Paulo, SP, Brazil. RN, Instituto do Coração, Hospital das Clínicas, Faculdade de Medicina, Universidade de São Paulo, São Paulo, SP, Brazil.; 3 PhD, Associate Professor, Escola de Enfermagem, Universidade de São Paulo, São Paulo, SP, Brazil.

**Keywords:** Nursing, Thinking, Concept Formation, Nursing Process, Decision Making

## Abstract

**Objective:**

to analyze the concept of critical thinking (CT) in Rodger's evolutionary perspective.

**Method:**

documentary research undertaken in the Cinahl, Lilacs, Bdenf and Dedalus databases, using the keywords of 'critical thinking' and 'Nursing', without limitation based on year of publication. The data were analyzed in accordance with the stages of Rodger's conceptual model. The following were included: books and articles in full, published in Portuguese, English or Spanish, which addressed CT in the teaching and practice of Nursing; articles which did not address aspects related to the concept of CT were excluded.

**Results:**

the sample was made up of 42 works. As a substitute term, emphasis is placed on 'analytical thinking', and, as a related factor, decision-making. In order, the most frequent preceding and consequent attributes were: ability to analyze, training of the student nurse, and clinical decision-making. As the implications of CT, emphasis is placed on achieving effective results in care for the patient, family and community.

**Conclusion:**

CT is a cognitive skill which involves analysis, logical reasoning and clinical judgment, geared towards the resolution of problems, and standing out in the training and practice of the nurse with a view to accurate clinical decision-making and the achieving of effective results.

## Introduction

The concept of critical thinking (CT) has, over the last two decades, been one of the most discussed in the area of the teaching and clinical practice of Nursing^1^ ^-^ ^2^.However, as a transversal domain, it extends to the various areas in which people work, from the most simple and routine, to the most complex and painstaking professional and academic tasks^3^ ^-^ ^4^. 

The literature on CT has its roots in two academic disciplines: philosophy and psychology^4^ ^-^ ^6^. However, one can also observe a third axis in the field of education^4^ ^,^ ^6^. In philosophy, one seeks to define the hypothetical critical thinker based on her qualities and characteristics^4^ ^,^ ^6^, while psychology seeks to describe the critical thinker based on her skills or actions^4^. In the ambit of education, there is a focus on the teaching and assessment of CT skills. As a result, there is a lack of consensus regarding the concept of CT in the literature, especially in the area of Nursing, which is influenced by these three axes. 

In the area of healthcare - including nursing - the aging of the population, the increase in the complexity of illnesses and consequently in the care required, as well as the demand for services, require a professional with skills in CT, problem resolution and decision-making, and who is capable of appropriately accessing information, leading to safe, efficacious clinical practice which is based in scientific evidence^3^ ^,^ ^7^ ^-^ ^8^. 

CT is an essential tool for the teaching and care practice of the nurse, and must not be confused with intelligence, but, rather, understood as a skill which can be learned^9^ ^-^ ^10^. This being the case, because of the importance of the need to train professional nurses able to think critically, the *Red Iberoamericana de Investigación en Educación en Enfermería* (RIIEE) addressed this issue in its multicentric investigation project^11^carried out in 16 countries in Ibero-America, this study being part of this investigation. 

Researching CT in conjunction with an international investigation network^11^ ^)^ encouraged the authors to undertake the present work, as, as has been observed in the literature in Nursing^4^ ^,^ ^7^ ^,^ ^11^ ^-^ ^12^, the term CT presents variations, causing frequent disagreements, which indicates the need for refining and clarifying the concept. As a result, this study's objective is to analyze the concept of critical thinking in the teaching and clinical practice of Nursing, in the perspective of Rodger's evolutionary model of concept analysis^13^. The rationale for this study of the conceptual analysis of CT, specific to Nursing, is that it promotes clarification for future studies in the ambit of these professionals' academic and clinical practice.

## Method

A documentary study^14^ ^)^ based on the evolutionary model of concept analysis proposed by Rodgers^13^. This model understands the concept as a reverberation of the phenomena, which are dynamic, as they change over time and have a direct relationship with the context of their use. They have an inductive, nonsequential and descriptive character, indicating systematic rigor, considering a definition which presents conceptual problems^13^. 

The process of analysis of the concept took place based on its substitute terms and related concepts, attributes, antecedents, and implications, and involves five stages^13^. In the first stage, the concept of interest was identified^13^, in the present study, CT, including the expressions and substitute terms used for the concept in the literature. 

In the second stage, undertaken in February 2015, the authors proceeded to consult with the Cinahl, Lilacs and Bdenf databases^13^, due to these being considered to be important in the context of the Brazilian and foreign scientific production, in the ambit of Nursing. Use was also made of the Dedalus database, for obtaining textbooks of Nursing from the Collective Catalog of the Libraries of the University of São Paulo. As a specific descriptor for CT was not found, the same was inserted in the search as a keyword, along with the descriptor 'Nursing'. The works were identified using the search strategy of *critical thinking* AND *nursing* and its variations for Portuguese and Spanish, without limiting the search by year of publication. A total of 529 works were located in the databases mentioned. 

In the third stage, for collection of the relevant data, identification of the attributes, and the contextual basis of the concept^13^, the following were defined as inclusion criteria: textbooks and articles of Nursing published in full, published in English, Portuguese or Spanish, and which addressed CT in the teaching and clinical practice of Nursing. The following were defined as exclusion criteria: duplicated studies and those which did not address aspects related to the concept of CT. 

In the fourth stage, for analysis of the data and identification of their characteristics^13^, firstly the reading of the titles of the works selected in the second stage was undertaken, based in the criteria of inclusion and exclusion. A total of 481 works was obtained. Next, the abstracts were read in order to select those which addressed the issue of CT in the context of Nursing. Based on this refinement, 47 works were selected, including both books and articles. Following individual and thorough reading of each text in full, a sample of 42 was defined for analysis, including books and articles. At this point, the authors proceeded to reading based on the guiding questions: what are the characteristics/attributes of CT? What are the conditions/means which viabilize CT? What are the consequences of CT? In this stage, through an inductive process, theattributes, antecedents and consequence of the concept, which appeared with the greatest frequency, were identified in the texts. The organization of the stages of the phenomenon analyzed took place through typing, with each characteristic being listed separately, according to the number of authors who addressed it. The data were organized in a Microsoft Office Excel(r) 2013 spreadsheet.

In the fifth stage, implications or hypotheses for continuity of the development of the concept were listed^13^.

## Results

**Figure 1:**
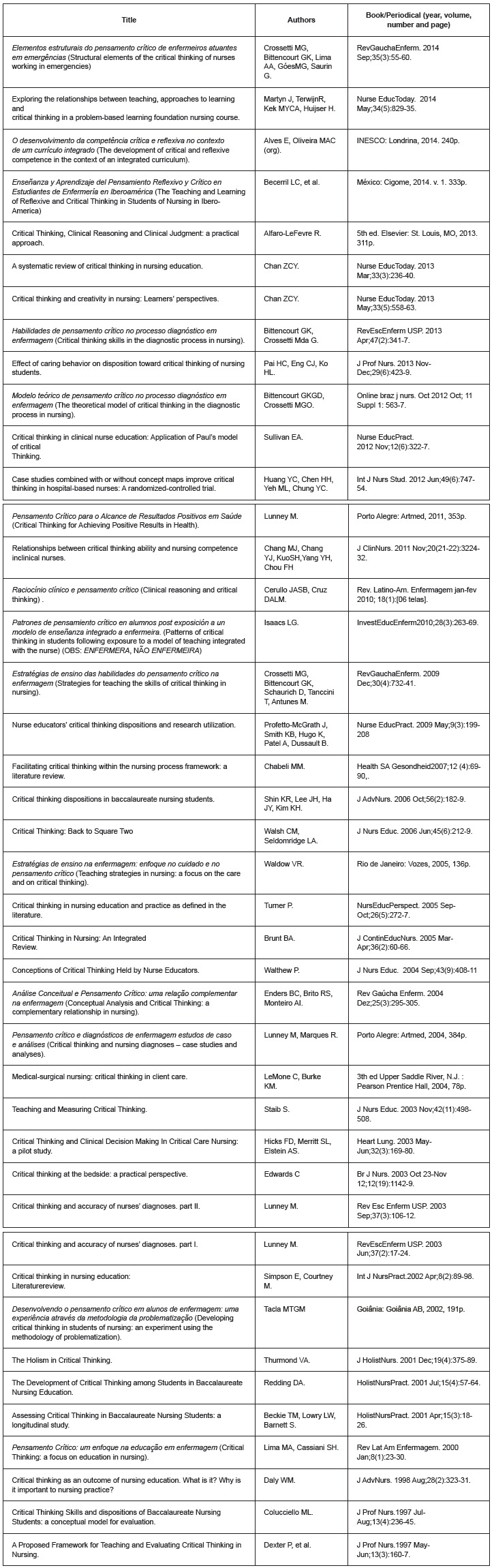
Works included for analysis, by title, authors and book or periodical. São Paulo, State of São Paulo (SP), Brazil, 2015.

Of the 42 works selected for analysis, eight were text books and 34 were articles, with 28 (66.6%) published in English, 12 (28.6%) in Portuguese, and two (4.8%) in Spanish. Of these, the oldest dated from 1997, and the most recent from September 2014: items were included by title, authors, and cataloguing data of the book or periodical (Figure 1). The results below were organized inductively, based on what Rodgers^13^proposes as substitute terms, the attributes, antecedents and consequences, and implications/hypotheses of the concept. 

The identification of the substitute terms represents the first approximation with the concept to be analyzed^13^, for understanding the origin, development, functions and interconnections of a concept with other similar words or expressions. The substitute terms for CT identified were: analytical thinking^15^ (14.3%), critical-creative thinking^16^(12%), nonlinear thinking^17^(9.5%) and critical-holistic thinking^16^ (9.5%). 

Differently from the substitute terms, the concepts related to CT (Table 1) have a link and closeness to it, but are not synonymous with it. 

**Table 1:** Factors/concepts related to Critical Thinking, by number of works analyzed. São Paulo, SP, Brazil, 2015

Related factors/concepts	N. of works	%
Decision-making	23	54.0
Clinical judgment	22	52.4
Reflexive thinking	20	47.6
Clinical reasoning	14	33.3
Problem resolution	10	23.8
Logical reasoning	7	16.6
Scientific method	7	16.6
Critical reflection	7	16.6
Diagnostic judgment	7	16.6
Diagnostic reasoning	5	12.0
Critical analysis	4	9.5
Critical assessment	4	9.5
Interpretive thinking	3	7.1
Socratic thinking	3	7.1
Complex reasoning	3	7.1

CT is characterized by higher-level thinking, which involves knowledge, experiences, dispositions (attitudes or habits of mind) and intellectual abilities. These essential characteristics, or attributes, were identified in the empirical material for analysis as skills and dispositions for CT, and are presented in Table 2. 

**Table 2:** Attributes (skills and dispositions) of the concept of Critical Thinking, by number of works analyzed. São Paulo, SP, Brazil, 2015

Attributes		N. of works	%
Skills			
	Analysis	39	92.8
	Assessment	34	80.4
	Inference	26	62.0
	Rational examination	23	54.7
	Interpretation	23	54.7
	Self-regulation	19	45.2
	Reflection	18	42.8
	Judgment	17	40.5
	Deduction	14	33.3
	Application of standards	10	23.8
	Questioning	9	21.4
	Summarizing	9	21.4
Dispositions			
	Open-mindedness	24	57.1
	Curiosity	23	54.7
	Honesty in facing personal prejudices	17	40.5
	Systematicity	16	38.0
	Clarity regarding questions and ideas	16	38.0
	Self-regulated judgment	16	38.0
	Actively searching for truth	15	35.7
	Maturity	13	31.0
	Metacognition	12	28.6
	Focus on accurate results	12	28.6
	Trusting in reason	12	28.6
	Seeking information	11	26.2
	Prudence in judgments	11	26.2
	Analyticity	10	23.8
	Perseverance	10	23.8
	Assessment of the credibility of the evidence	10	23.8

The antecedents, like the consequences of a concept, are conditioned to a contextual basis, in this case, the concept of CT in Nursing. In addition to this, they are related to the situational, temporal, sociocultural and course-related contexts of the profession at the present time^13^. The antecedents are considered the events, situations or phenomena which precede the concept investigated, while the consequences are situations which result from the occurrence of the concept in question (Table 3). 

**Table 3:** Antecedents and Consequences of the concept of Critical Thinking by number of works analyzed. São Paulo, SP, Brazil, 2015

Antecedents and Consequences		N. of works	%
Antecedents			
	Teaching in Nursing/Training of the Student of Nursing	25	59.5
	Teaching Policies/Curricular Guidelines of Nursing courses	23	54.7
	The need for better results in clinical practice	19	45.2
	Scientific and Technological Advances/Complexity of the health systems	18	42.8
	Cognitive, attitudinal and instrumental development of the nurse	8	19.0
	The need for decision-making/Management in health	8	19.0
	Practice Based in Scientific Evidence	7	16.6
	Safety in the care/quality of the health services	6	14.3
	Requirements of the job market	5	12.0
	Reflection, analysis and criticism	7	16.6
	Actions based in relevant evidence	5	12.0
	Summary	9	21.4
Consequences			
	Clinical decision-making	37	88.0
	Clinical judgment	23	54.8
	Clinical reasoning	22	52.4
	Organization of the Nursing Process	18	42.8
	Problem resolution	15	35.7
	Better results in the practice of teaching	11	26.2
	Diagnostic accuracy	9	21.4
	Positive results in the attendance of the patient	8	19.0
	Reflection, analysis and criticism	7	16.6
	Actions based in relevant evidence	5	12.0

The implications and hypotheses represent, respectively, the results and possibilities to be achieved^13^by a critical thinker in the ambit of Nursing and are important aspects for the development of the concept over time^13^. In accordance with the frequency as certained in the works, the implications and hypotheses are listed in Table 4. 

**Table 4:** Implications and hypotheses of the concept of Critical Thinking by number of works analyzed. São Paulo, SP, Brazil, 2015

Implications and Hypotheses	N. of works	%
Achieving effective results in care for the patient, family and community	15	35.7
Safety and quality of the care	14	33.3
Professional growth and satisfaction	13	31.0
Autonomy in the work process	13	31.0
Achieving effective results in the teaching and in the routine of the nursing practice	10	23.8
Citizens with critical-creative spirit in the social milieu which they are part of	8	19.0
Development of competencies and skills which go beyond the technical dimension	8	19.0
Establishment of interdisciplinary connections	4	9.5

## Discussion

### Substitute terms and related factors/concepts 

The concept of CT remains little clarified in the Nursing literature^1^ ^,^ ^16^, it being possible to identify a number of close words/expressions which are capable of representing or even explaining attributes and skills of an ideal critical thinker. In this regard, it becomes important to make it clear that substitute terms figure as expressions with meanings which are similar to the concept of CT. This aspect was discussed in one review study^16^, which indicated that clinical reasoning and CT are, often, used as synonymous terms inappropriately, as CT involves skills and attitudes which are necessary for the development of clinical reasoning.

Based on the empirical material researched, the most frequent substitute term was analytical thinking, followed by critical-creative thinking. These substitute terms, to the contrary of clinical reasoning, are synonyms, as they present a semantic interconnection with CT. As a result, when a nurse assesses a patient's health problem, observing, examining, recognizing and raising hypotheses regarding health problems, she is using analytical thinking, which can also be termed CT^17^.

On the other hand, the related factors (Table 1) have a correlation of cause or effect with the concept. For CT, the main related factors identified are aptitudes which are necessary for undertaking the stages of the Nursing process, such as decision-making^16^, clinical judgment^18^ ^-^ ^19^ ^)^ and clinical reasoning^11^. This relationship is not one of mere causality, it having been demonstrated in one study^20^ that CT skills establish a relationship between themselves and, in the same way, with the stages of the diagnostic process in Nursing. Furthermore, CT involves skills and attitudes which are fundamental for achieving excellent goals of diagnostic accuracy. It is emphasized that, as a structural element of CT, clinical reasoning leads to clinical judgment, resulting in clinical decision-making^12^ ^,^ ^15^ ^,^ ^20^. 

Another term which has been described many times coupled with CT, nearly forming a single term, is the concept of reflexive thinking^11^ ^,^ ^15^ ^-^ ^16^. The reasoning for this may be related to the fact that some authors consider reflection to be a skill of the critical thinker. Based on the hypothesis that reflection leads to critical thinking, one experimental study^21^, using reflexive writing, was undertaken with 70 undergraduate students of nursing. As a result, a significant increase was observed in the CT skills among the students of the experimental group. It is, therefore, relevant to undertake studies of this issue, as they assist in the process of understanding and evolution of the concepts. 

Generally speaking, the identification of the substitute terms and related factors, respectively, made it possible to broaden the list of synonymous concepts and those which are interrelated. As a result, the related concepts allow the correct application of words which contain within themselves similar philosophical presuppositions, in this case, CT. Besides this, they assist in the understanding of the application of the concept of CT in the context of the practice of the student or nurse, and its importance for diagnostic reasoning, clinical judgment and efficacious decision-making. 

### Attributes of the concept of CT 

The attributes bring together a real definition of the concept, different from the nominal definition provided in dictionaries, which simply substitute one word or expression with another which is synonymous^13^. As a result, the attributes are an integral part of a concept, corresponding to its characteristics. The sum of these attributes typifies and characterizes the concept. As presented in Table 2, the attributes which qualify CT are made up of skills and dispositions. 

It is inferred, based on these results, that in the literature there is a major influence of the Delphi Report^22^, a consensus undertaken at the beginning of the 1990s by an interdisciplinary group of specialists in CT from the area of the humanities, social sciences, and education, defining the essential characteristics of the ideal critical thinker. The report^22^ ^)^ describes a significant proportion of the attributes identified in this study, such that these constitute the characteristics examined in most of the psychometric scales for assessing the development of CT. 

In the literature analyzed, the skill mentioned most frequently for a critical thinker was "analysis"^11^ ^,^ ^15^ ^,^ ^19^ ^,^ ^22^ ^)^ or the ability to break something down in order to achieve an understanding of a given situation. It is believed that this skill is essential in academic and professional activity in Nursing, in that the understanding of the whole is limited to analysis of the parts, whether in the learning of a technique or in taking the patient history.

The most frequent disposition was "open-mindedness"^19^ ^,^ ^22^ ^)^ or impartiality in assessment, overcoming preconceptions in a rational way for prudent judgment and later decision-making. However, as studies indicate^4^ ^,^ ^6^ ^,^ ^20^, these skills and dispositions must be constantly exercised, given that the human mind tends to base its decisions on patterns of the cause-and-effect type, often intuitively, without careful examination of the possible alternatives. 

As a result, for the improvement in the attributes of CT, the student or professional of Nursing must adopt a stance of seeking, in a careful, rational and active way, the improvement of the cognitive and affective aspects which are inherent to her professional and personal activities^21^ ^,^ ^23^. Based on this definition, it may be perceived that CT is a practical activity based on the sensible search for reason, through skills and dispositions, it being essential for the individual to have an inclination or attitude for carrying these out^20^. In this process, the importance of thinking about thought (metacognition)^4^ ^)^ is underlined as a necessary path to developing CT skills. 

### Antecedents of the Concept 

Based on the categories presented in Table 3, the emphasis on the development of CT in the teaching and training of the student of Nursing is clear^11^ ^,^ ^16^, as well as the importance of the Teaching Policies, in particular the Brazilian National Curricular Guidelines (DCNs)of the undergraduate courses in Nursing^11^ ^,^ ^24^ ^-^ ^25^ ^)^ as phenomena which have leveraged the discussions and highlighted the importance of training nurses in the skill of thinking critically. 

In Brazil, the DCNs^25^ ^)^ bestow curricular flexibility, which viabilizes pedagogical projects geared towards specific goals or demands. As a result, there is the possibility of a teaching permeated by CT, as it progresses towards problematizing training, linking teaching, work and community. 

Another aspect also evidenced as a predecessor of CT was the seeking of better results in clinical practice^14^ ^,^ ^18^ ^,^ ^22^ ^)^ and the scientific and technological advance^25^ ^-^ ^26^, which represent an important challenge at the time of writing, as a result of the competencies and skills which are necessary and required for the students and nurses in order to respond efficaciously to the complexity of the health systems. In this regard, studies^16^ ^,^ ^19^ ^,^ ^23^indicate that the rapid technological and scientific advances in the area of health, added to the rapid expansion of Nursing's body of knowledge, have required critical thinkers in the profession. 

The challenge of training a nurse who is capable of acting in a way that responds to the demands of a society in constant transformation is closely related to the need for cognitive, attitudinal and instrumental development so as to seek better results in practice and in health management^15^ ^,^ ^23^ ^,^ ^27^. Besides this, the nurse's decision-making must take into account the evidence deriving from professional experience, the scientific literature and the patient's requirements, with a view to the safety and quality of the health services. 

### Consequences of the concept of CT 

The consequences of a concept are closely related to the antecedents and must follow them^13^. As a result, the consequences presented in Table 4 are considered to be situations which result from the occurrence of CT. Hence, they trigger actions in practice and/or teaching. 

The consequence identified with the highest frequency was "clinical decision-making"^7^ ^,^ ^23^, followed by "clinical judgment"^11^ ^,^ ^19^ ^-^ ^20^ ^)^ and "clinical reasoning"^12^ ^,^ ^15^. The latter two, although distinct, are fundamental in the nurse's practice and lead to clinical decision-making. Clinical reasoning involves mental processes applied in the activities of the nurse who, based on a judgment based on knowledge, will take an appropriate decision. In this process of intellectual action, which results in clinical intervention, CT acts as a regulator of the patterns of thinking^8^ ^,^ ^12^. It thus covers an attitude of being disposed to considering, in a conscious way, the problems which arise in the ambit of the clinical experience or of teaching. 

For effective decision-making based in CT, therefore, persistent effort is required for examining any situation or supposed form of knowledge in the light of evidence which supports new conclusions in regard to it^10^ ^,^ ^15^. One study^23^ ^)^ which assessed the structural elements of CT in 20 nurses from emergency services corroborated these findings, as it emphasized the importance of CT for clinical decision-making, as well as its interconnection in the managerial, multi-professional and teaching ambits. It is relevant to emphasize that the first three consequent terms identified previously also appeared as related concepts in Table 1, reinforcing the tenuous interconnections with CT. 

### Implications and hypotheses of the concept of CT 

The main implications and hypotheses listed in Table 4 are related to the nurse's care practice. As a result, the most frequent implications for a critical thinker in Nursing were: achieving effective results in care to the patient, family and community^19^ ^,^ ^23^ (35.7%) and safety and quality of the care^12^ ^,^ ^16^ ^,^ ^23^ (33.3%). Therefore, it is believed that the structural elements of CT (skills and dispositions), continuously exercised in the ambit of nursing, entail effective and safe care. It is inferred that CT can be a mediating instrument in the improvement of reasoning and the exercise of practice, conferring upon the nurse competences and skills for diagnosing accurately. 

For this, it is important to consider CT as a process of dynamic and continuous improvement which can be learnt by the nurse. As a result, the improvement of reasoning skills, the constant search for new knowledge, the aim of solving problems and issuing judgments is required, enabling the nurse to predict, diagnose and validate the conclusions obtained, always reviewing these critically^19^ ^,^ ^26^ ^-^ ^27^.

One longitudinal Australian study^28^ ^)^ assessed CT skills in 134 students, at two points: upon entering the undergraduate course in Nursing, and three years later, after graduating, using the Health Sciences Reasoning Test. It was concluded that the students significantly increased their CT scores after progressing through the course, principally improving their abilities in analysis and deduction. The students who had the highest scores in the test had better academic performance. These results corroborate the importance of the undergraduate course in helping the students to progress in their CT skills. 

As indicated by the works analyzed^15^ ^,^ ^23^ ^,^ ^26^, it is believed that, as she progresses in the dimensions of CT, the student or nurse can achieve greater professional satisfaction and autonomy in her work process. Among various factors analyzed in one study^29^, the nurses reported that they achieved greater professional satisfaction as they achieved greater autonomy. In this regard, CT and its structural elements are intermediary tools which lead the professional to assertiveness in her clinical actions, creating safety, autonomy and satisfaction. 

## Conclusion

The concept analysis, based in Rodger's evolutionary model, clarified the phenomenon and allowed a correct understanding of CT, with implications for education and care in Nursing. 

As antecedents of the concept, emphasis was placed on the need for training in critical thinking while still on the undergraduate course, with a consequent search for better results in clinical practice. This being the case, the main concepts related to CT are those which make up the stages of the Nursing Process, such as decision-making, clinical judgment, and clinical reasoning. 

As a result, the understanding of the related factors, attributes, antecedents and consequences of CT made it possible to infer CT as a cognitive skill which involves a process of analysis, logical reasoning and clinical judgment, geared towards problem resolution. From this perspective, it has been emphasized in the training and care practice of the nurse, with a view to accurate clinical decision-making and to achieving effective results in the Nursing interventions for the patient, family and community.

As limitations of the study, the fact is highlighted that studies were selected from Latin American databases, with the exception of Cinahl. However, the study met the needs of the multicentric RIIEE project. As a consequence, broader studies become important, considering that the concept changes over time. Furthermore, it is necessary to develop the clarification of the concept of CT in other areas of knowledge.

## References

[1] Tajvidi M, Ghiyasvandian S, Salsali M. (2014). Probing concept of critical thinking in nursing education in Iran: a concept analysis. Asian Nurs Res. (Korean Soc Nurs Sci).

[2] Azizi-Fini I, Hajibagheri A, Adib-Hajbaghery M. (2015). Critical Thinking Skills in Nursing Students: a Comparison Between Freshmen and Senior Students. Nurs Midwifery Stud.

[3] Pitt V, Powis D, Levett-Jones T, Hunter S. (2015). The influence of critical thinking skills on performance and progression in a pre-registration nursing program. Nurse Educ Toda.

[4] Abrami PC, Bernard RM, Borokhovski AW, Surkes MA, Tamim R, Zhang D (2008). Instructional interventions affecting critical thinking skills and dispositions: A stage 1 meta-analysis. Rev Educ Res.

[5] Bacanlia H, Dombaycib MA, Demirc M, Tarhand S. (2011). Quadruple Thinking: Creative Thinking. Procedia Soc Behav Sci.

[6] Almeida LS, Franco AHR. (2011). Critical thinking: Its relevance for education in a shifting society. Rev Psicol.

[7] Papathanasiou IV, Kleisiaris CF, Fradelos EC, Kakou K, Kourkouta L. (2014). Critical thinking: the development of an essential skill for nursing students. Acta Inform Med..

[8] Huang GC, Newman LR, Schwartzstein RM. (2014). Critical thinking in Health Professions Education: Summary and Consensus Statments of the Millennium Conference 2011. Teach Learn Med.

[9] Ozkahraman S, Yildirim B. (2011). An overview of critical thinking in nursing and education. Am Int J of Contemp Res.

[10] Paul SA. (2014). Assessment of critical thinking: a Delphi study. Nurse Educ Today.

[11] Becerril LC, Gomez MAJ, Püschel VAA, Fierros GA, Porras MDB, Isaacs LG (2014). Enseñanza y aprendizaje del pensamiento reflexivo y crítico en estudiantes de enfermería en Iberoamérica.

[12] Jensen R, Cruz DALM, Tesoro MG, Lopes MHBM. (2014). Translation and cultural adaptation for Brazil of the Developing Nurses' Thinking model. Rev. Latino-Am. Enfermagem.

[13] Rodgers BL (2000). Concept analysis: An evolutionary view. In Rodgers BL, Knafl KA. Concept development in nursing: foundations, techniques, and applications. Saunders. 2nd Ed.

[14] Marconi MA, Lakatos EM (2010). Fundamentos de metodologia científica.

[15] Cerullo JASB, Cruz DALM. (2010). Clinical reasoning and critical thinking. Rev. Latino-Am. Enfermagem.

[16] Chan ZCY (2013). Critical thinking and creativity in nursing: Learners' perspectives. Nurse Educ Today.

[17] Bittencourt GKGD, Crossetti MGO. (2013). Critical thinking skills in the nursing diagnosis process. Rev Esc Enferm USP.

[18] Turner P. (2005). Critical thinking in nursing education and practice as defined in the literature. Nurs Educ Perspect.

[19] Brunt BA (2005). Critical thinking in nursing: an integrated review. J Contin Educ Nurs.

[20] Bittencourt GKGD, Crossetti MGO. (2013). Critical Thinking skills in the nursing diagnosis process. Rev Esc Enferm USP.

[21] Naber J, Wyatt TH. (2014). The effect of reflective writing interventions on the critical thinking skills and dispositions of baccalaureate nursing students. Nurse Educ Today.

[22] Facione PA (1990). Critical thinking: A statement of expert consensus for purposes of educational assessment and instruction..

[23] Crossetti MGO, Bittencourt GKGD, Lima AAA, Góes MGO, Saurin G. (2014). Structural elements of critical thinking of nurses in emergency care. Rev Gaucha Enferm.

[24] Martyn J, Terwijn R, Kek MYCA, Huijser H. (2014). Exploring the relationships between teaching, approaches to learning and critical thinking in a problem-based learning foundation nursing course. Nurse Educ Today.

[25] Ministério da Saúde (BR), Conselho Nacional de Educação, Câmara de Educação Superior (2001). Resolução CNE/ CES nº 3, de 7 de novembro de 2001: institui Diretrizes Curriculares Nacionais do Curso de Graduação em Enfermagem.

[26] Pucer P, Trobec I, Zvanut B. (2014). An information communication technology based approach for the acquisition of critical thinking skills. Nurse Educ Today.

[27] Carley A. (2015). Using technology to enhance nurse practitioner student engagement. Nurse Pract.

[28] Pitt V, Powis D, Levett-Jones T, Hunter S. (2015). The influence of critical thinking skills on performance and progression in a pre-registration nursing program. Nurse Educ Today.

[29] Siqueira VTA, Kurcgant P. (2012). Job Satisfaction: a quality indicator in nursing human resourse management. Rev Esc Enferm USP.

